# Supplementary material for: Antibacterial Effect of Zinc Oxide-Based Nanomaterials on Environmental Biodeteriogens Affecting Historical Buildings
Source: Nanomaterials (Basel). 2020 Feb 16;10(2):335. doi: 10.3390/nano10020335 (PMC7075178; doi:10.3390/nano10020335)
Supplement: Supplementary file 1 [file nanomaterials-10-00335-s001.pdf]

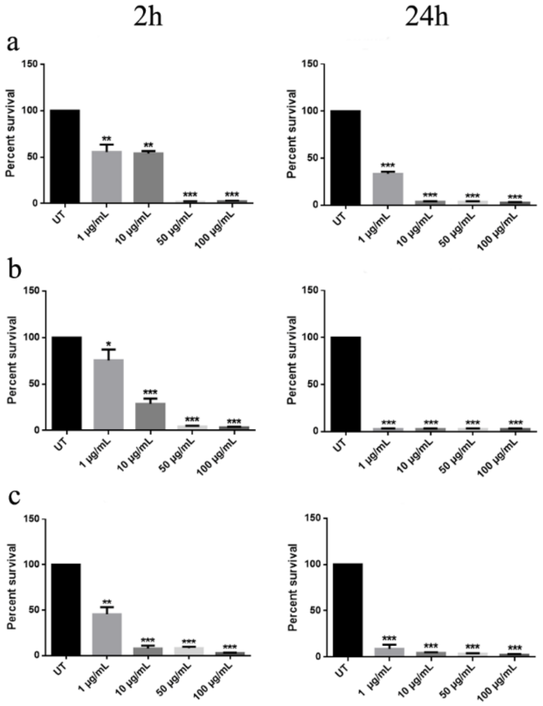

Figure S1. Cell viability after exposure to different concentrations of ZNGs. Treatments were repeated after 2 hours and after 24 hours; then CFU were counted. (a), (b) and (c) indicate respectively *A. aurescens* TC4, *A. spanius* TC1 and *A. spanius* TC7. To evaluate statistical significance a one-way ANOVA analysis with the Bonferroni post-test was used (*p < 0.5, **p < 0.01 and ***p < 0.001 with respect to UT).

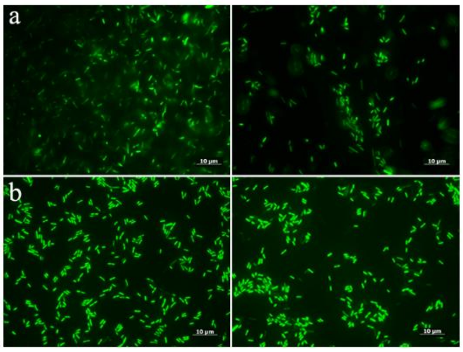

Figure S2. LIVE/DEAD staining after treatment with ZNGs. Fluorescence microscope images of untreated bacterial strains (on the left) and treated cells with a concentration of ZNGs of 10 µg/mL. (a) and (b) indicate respectively *A. aurescens* TC4 and *A. spanius* TC7.
